# A Rare Case of a Balo's Concentric Sclerosis-Like Lesion in a Young Adult Woman

**DOI:** 10.7759/cureus.46803

**Published:** 2023-10-10

**Authors:** Kailee Vickaryous, Fernando Poli De Frias, George A Gonzalez

**Affiliations:** 1 Dr. Kiran C. Patel College of Osteopathic Medicine, Nova Southeastern University, Fort Lauderdale, USA; 2 Internal Medicine, Mount Sinai Medical Center, Miami, USA; 3 Neurology, Mount Sinai Medical Center, Miami, USA

**Keywords:** balo's concentric sclerosis, relapsing-remitting, neurologic symptoms, brain mri, neurodegenerative disorders, demyelinating disorders, multiple sclerosis

## Abstract

Balo's concentric sclerosis (BCS) is a rare demyelinating disorder of the central nervous system (CNS). Distinguishing BCS from other demyelinating disorders such as multiple sclerosis (MS) or from neoplasms can be difficult clinically; however, MRI aids in the identification of the disease. We describe the case of a 37-year-old female presenting with sudden onset of neurologic symptoms associated with a solitary rounded white-matter lesion suggestive of BCS. This rare disorder can present with heterogenous symptoms, imaging findings, and response to treatment. Furthermore, more in-depth analysis of the presentations and treatment outcomes of BCS are necessary in order to create a more robust plan of care.

## Introduction

Balo's concentric sclerosis (BCS) is a rare demyelinating disorder of the central nervous system (CNS) that was first described by a pathologist named Joseph Balo in 1928 [1]. The term “concentric sclerosis” derives from the disorder’s pattern of circular areas with alternating damaged myelin and undamaged myelin in various regions of the brain and spinal cord.

Previously, BCS was diagnosed through visualization of its unique demyelinating lesions upon autopsy. However, current practices detect BCS with ante-mortem methods such as MRI [2]. This disorder shares features with other demyelinating diseases and is generally considered a rare variant of multiple sclerosis (MS). However, this association has been under debate.

While MS is one of the most common neurodegenerative disorders across the world with an estimate of 2.8 million living with the disease worldwide in 2020, the amount of BCS cases reported has not been entirely quantified [3]. One retrospective analysis conducted in 2018 reported finding 132 total cases in medical literature from the years 1980 to 2017 [4]. The cause of the disease is unknown. Attacks from this disease can occur rapidly over weeks to months without remission or it can take a relapsing course with periods of symptoms followed by disappearance of symptoms [5,6]. Some have hypothesized that the initial demyelination creates a hypoxic response leading to creation of an edge of demyelination, leading to the alternations of myelinated and demyelinated areas [7].

Previously, the clinical course of BCS was thought to resemble other variants of MS such as Margbug’s or Schilder’s, both of which take a fulminant course with often fatal outcomes. More recent studies highlight how BCS can also take a more benign course when there is prompt identification and treatment [1]. This case study supports that prolonged survival and resolution of neurologic symptoms can be achieved with prompt treatment.

## Case presentation

A 37-year-old woman of northern European descent with medical history of juvenile idiopathic arthritis (JIA), presented to the emergency department with sudden onset of “whole body pain”, blurry vision, followed by weakness of the left hemibody. The patient reported that five days prior, she experienced a similar sudden onset of “whole body pain” associated with chest pressure that woke her up from sleeping. She experienced dizziness and blurry vision when getting out of bed that evening. She was unable to drink water due to difficulty bringing the glass towards her mouth. Despite mild improvement upon waking up, she continued having tongue weakness and difficulty with speech that morning. After a second episode, she decided to go to the emergency department for further workup. 

Upon arrival, the patient was alert and oriented but anxious-appearing. Additionally, she complained of mental fogginess, short-term memory loss and depression over the last year. She denied any motor and sensory symptoms in the past and reported no prior episodes of blurred vision. She also denied tobacco, alcohol, or drug abuse. The patient was hemodynamically stable with normal vitals. On neurological examination, the patient was alert and oriented to person, place, time, and event. On assessment of the cranial nerves, the patient had left nasolabial flattening. On motor examination, the patient had subtle weakness in the left hemibody. Sensation and cerebellar function was intact. No tremors or involuntary movements were present. Rest of physical exam was unremarkable. Laboratory workup was negative for leukocytosis (Table 1). Cerebrospinal fluid (CSF) results were normal (Table 2). CSF gram stain, acid fast bacilli stain, protein, glucose, bacterial culture, herpes simplex virus (HSV) 1 & 2 and varicella zoster polymerase chain reaction (PCR), venereal disease research laboratory test (VDRL), and oligoclonal bands were negative. Serum aquaporine-4 antibody titers and angiotensin-converting enzyme were negative (Table 3).

**Table 1:** Summary of laboratory workup for the patient at admission CBC: Complete blood count; WBC: White blood cell count; RBC: Red blood cell count; MCV: Mean corpuscular volume; MCH: Mean corpuscular hemoglobin; MCHC: Mean corpuscular hemoglobin concentration; CO_2_: carbon dioxide; BUN: Blood urea nitrogen; EGFR: Estimated glomerular filtration rate; HDL: High density lipoprotein; LDL: Low density lipoprotein

Laboratory blood tests	Normal Ranges	Patient’s Results
CBC		
WBC	(4.5-11.0 x 10^3^/mm^3^)	5.17
RBC	(3.9-5.8 x 10^6^/µL)	4.47
Hemoglobin	(13-18 g/dl)	14.2
Hematocrit	(37.0-49.0%)	41.0
MCV	(78-100 µm^3^)	91.7
MCH	(25.0-35.0 pg/cell)	31.8
MCHC	(31-37 g/dl)	34.6
Platelets	(130-400 x 10^3^/µL)	231
%Neutrophils	(45-75%)	68.8
%Lymph	(16-46%)	1.2
%Mono	(4-11%)	0.34
%Eos	(0-5%)	0.02
%Baso	(0-3%)	0.03
%Immature Granulocytes	(<1%)	0.02
Metabolic Profile		
Sodium	(135-145 mmol/L)	140
Potassium	(3.4-5.0 mmol/L)	4.2
Chloride	(95-108 mmol/L)	105
CO_2_	(20-32 mmol/L)	27.9
BUN	(8-25 mg/dL)	7.0
Creatinine	(0.8-1.1 g/day)	0.80
EGFR	(>60 mL/min/1.75 m^2^)	>60
Glucose	(70-100 mg/dL)	101
Calcium	(8.5-10.5 mg/dL)	8.6
Albumin, serum	(3.5-4.9 mg/dL)	4.5
Lipid Profile		
Cholesterol	(<200)	213
Triglyceride	(<150)	43
HDL	(40-60)	83
LDL	(<160)	121

**Table 2:** Summary of CSF values RBC: Red blood cell count; CSF: Cerebrospinal fluid

Test	Normal Ranges	Patient’s Results
Color		Colorless/clear
Xanthochromia		Absent
RBC	(<1 cells/ µL)	6
Protein	(15-45 mg/dL)	38
Glucose	(40-70 mg/dL)	68
Gram Stain		No neutrophils or organism
CSF Bacterial Culture		No growth

**Table 3:** Additional results CSF: Cerebrospinal fluid; IgG: Immunoglobulin G; HSV: Herpes simplex virus; PCR: Polymerase chain reaction; VDRL: Venereal disease research laboratory test; VZV: Varicella zoster virus; QUAL: Qualitative

Test	Normal Ranges	Patient’s Results
Albumin, CSF	(5-34 mg/dL)	20.7
IgG, CSF	(0-4.5 mg/dL)	2.6
IgG index, CSF	(0.32-0.60)	0.46
Serum-IgG	(620-1520 mg/dL)	1210
Oligoclonal Bands, CSF	(No Bands)	Absent
Anti-aquaporin 4 antibodies, Serum		Negative
HSV1 PCR, CSF		Not detected
HSF2 PCR, CSF		Not detected
VZV DNA QUAL PCR, CSF		Not detected
VDRL, CSF		Non-reactive

**Figure 1:**
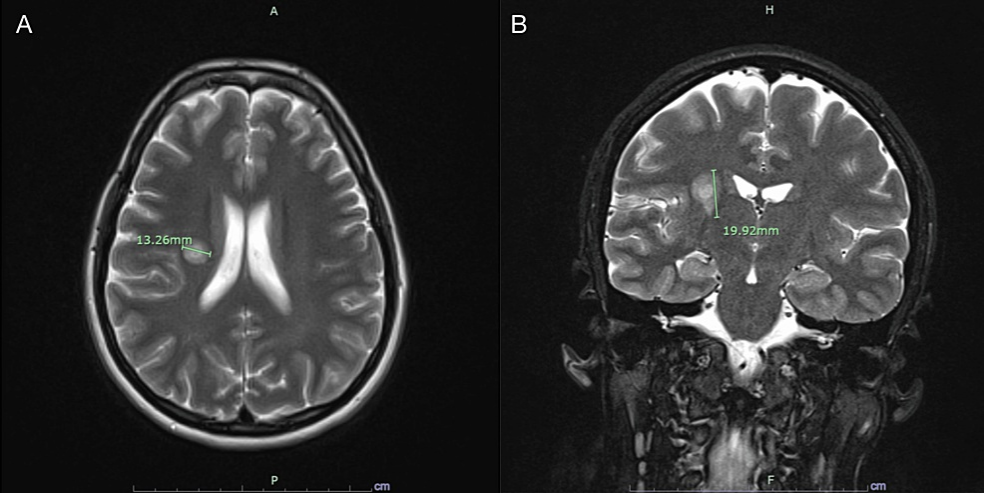
Initial MRI MRI brain demonstrating a solitary BCS-type lesion. Figure 1A displays a T2-weighted axial image showing a well-defined, rounded lesion measuring 13.26 mm in the right corona radiata with alternating layers of high and low signal intensity giving a “bullseye” or “onion bulb” appearance without any significant perilesional edema. Figure 1B displays a T2-weighted coronal image of the same lesion measuring 19.92 mm.

CT of the brain showed a well-defined, hypo-dense round lesion (~1.3 cm) with possible mild mass effect in the right corona radiata. The brain MRI revealed the lesion had T2/flair hyperintense signal with a bullseye appearance, concerning for BCS (Figure 1). Cervical, thoracic, and lumbar spine MRIs were negative. The patient was diagnosed with BCS by exclusion by the neurology and internal medicine teams. 

**Figure 2:**
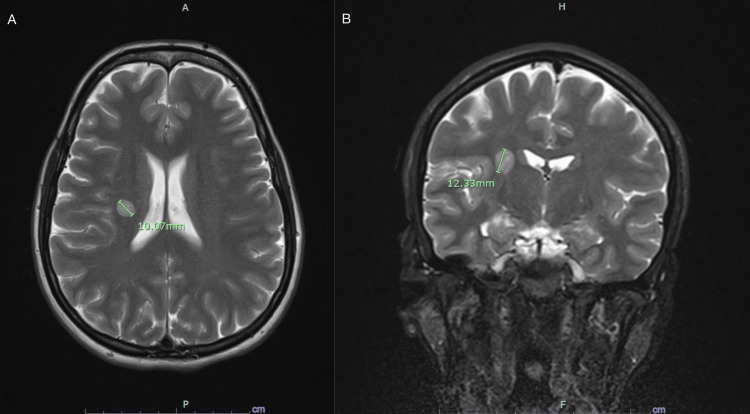
Follow-up MRI four months later Follow-up MRI brain revealing a decrease in size of the previously described lesion in the right corona radiata. Figure 2A displays a T2-weighted axial image showing a decrease in size to 10.07 mm. The coronal view in Figure 2B reveals a decrease in size to 12.33 mm.

Treatment was initiated with high-dose intravenous methylprednisolone (IVMP) 1 gram given once daily for 5 days. Patient was discharged home, given clinical improvement. Follow-up MRI four months later revealed a decrease in size and enhancement of the lesion with an unremarkable neurological exam with resolution of the left nasolabial flattening and subtle weakness in the left hemibody (Figure 2).

## Discussion

Cases of BCS are rare and can be difficult to diagnose due to the heterogeneity of symptoms. This disease presents similarly to MS and can affect the brain, the spinal cord, and the optic nerve. The first symptoms often include losing vision in one eye, loss of strength in an arm or a leg, or onset of numbness in the extremities. With our patient’s symptoms of blurry vision and weakness of the left hemibody, onset of MS was initially highly suspected. This case illustrates the need for distinction between rare demyelinating disorders such as BCS and more common disorders such as MS. 

Four different disease courses of MS have been described, including clinically isolated syndrome (CIS), relapsing-remitting MS, secondary progressive MS, and primary progressive MS. CIS represents the first clinical onset of symptoms suggestive of MS [8]. In our patient’s case, the potential for CIS was investigated as this was the first time she experienced symptoms suggestive of CNS demyelination. Because there is no single diagnostic test or clinical symptom to diagnose a patient with CIS, diagnostic criteria from the International Panel of McDonald and colleagues was used to determine whether the single episode of CIS might progress. Early diagnosis is important, because while some patients might only experience one clinical episode, a majority of patients proceed to develop the course of relapsing-remitting MS.

The diagnosis of MS has been outlined by the 2017 McDonald criteria, which states that an individual must have evidence of CNS damage that is disseminating in time and space. Disseminating in time (DIT) means that there is evidence of neurological damage occurring over time, and disseminating in space (DIS) means that the demyelination affects multiple regions of the nervous system. This is often demonstrated by either a second clinical episode or the appearance of a new lesion detected on MRI. The MRI of our patient’s demyelinating lesion demonstrated that it was neither expanding nor disseminating, which distinguished it from the DIT and DIS criteria required to diagnose MS. 

A striking difference under recent investigation is the lack of oligoclonal bands found in the CSF of BCS patients. Over 95% of MS patients are found to have oligoclonal bands in their CSF, which are usually detectable throughout the course of the disease, whereas a study in 2018 that conducted a retrospective analysis of 146 lumbar punctures of recorded BCS patients found that 66% of the total cases lacked oligoclonal bands in the CSF [4]. Our patient’s negative oligoclonal bands in addition to the “onion bulb” lesion discovered on MRI suggested that her condition was a demyelinating disorder distinct from MS. BCS physiopathology is unclear, although several mechanisms have been proposed [9]. A link between autoimmune disorders, such as JIA, and demyelination has been reported, but predisposing factors remain unclear [10,11]. 

Therapeutic trials for BCS have not been conducted in the past due to its rarity. Furthermore, the first-line treatment for MS has been utilized for most BCS cases. This treatment course includes the use of corticosteroids during the active phase of the disease. Marked clinical improvement from the initial attack is reported in most patients after IVMP, however, BCS can course as a relapsing-remitting syndrome, requiring further interventions [12]. Of note, about 85% of MS patients have relapsing-remitting MS[3]. The amount of relapsing-remitting BCS patients has not been quantified, and there is currently no standard recommendation regarding maintenance therapy. Furthermore, close follow-up monitoring is required in all cases of BCS. 

## Conclusions

Patients with BCS present with heterogeneity of symptoms. Therefore, it is imperative that these patients receive a proper workup attentive to clinical presentation, imaging, and CSF findings to characterize the disorder and differentiate it from MS as well as other degenerative disorders. Follow-up is imperative to assess for DIT, DIS criteria, and response to therapy. Ultimately, this rare disorder warrants clearer diagnostic and treatment guidelines that could ultimately optimize outcomes and improve the quality of life of BCS patients.
